# A Dietary Cholesterol-Based Intestinal Inflammation Assay for Improving Drug-Discovery on Inflammatory Bowel Diseases

**DOI:** 10.3389/fcell.2021.674749

**Published:** 2021-06-03

**Authors:** Nuno-Valério Silva, Diogo Carregosa, Catarina Gonçalves, Otília V. Vieira, Cláudia Nunes dos Santos, António Jacinto, Carolina Lage Crespo

**Affiliations:** ^1^iNOVA4Health, CEDOC, NOVA Medical School, NMS, Universidade Nova de Lisboa, Lisbon, Portugal; ^2^Instituto de Biologia Experimental e Tecnológica, Oeiras, Portugal

**Keywords:** inflammatory bowel disease, dietary cholesterol, drug screening and discovery, innate immunity, myeloid cells, dietary phenolic acids, inflammation and immunity

## Abstract

Inflammatory bowel diseases (IBD) with chronic infiltration of immune cells in the gastrointestinal tract are common and largely incurable. The therapeutic targeting of IBD has been hampered by the complex causality of the disease, with environmental insults like cholesterol-enriched Western diets playing a critical role. To address this drug development challenge, we report an easy-to-handle dietary cholesterol-based *in vivo* assay that allows the screening of immune-modulatory therapeutics in transgenic zebrafish models. An improvement in the feeding strategy with high cholesterol diet (HCD) selectively induces a robust and consistent infiltration of myeloid cells in larvae intestines that is highly suitable for compound discovery efforts. Using transgenics with fluorescent reporter expression in neutrophils, we take advantage of the unique zebrafish larvae clarity to monitor an acute inflammatory response in a whole organism context with a fully functional innate immune system. The use of semi-automated image acquisition and processing combined with quantitative image analysis allows categorizing anti- or pro-inflammatory compounds based on a leukocytic inflammation index. Our HCD gut inflammation (HCD-GI) assay is simple, cost- and time-effective as well as highly physiological which makes it unique when compared to chemical-based zebrafish models of IBD. Besides, diet is a highly controlled, selective and targeted trigger of intestinal inflammation that avoids extra-intestinal outcomes and reduces the chances of chemical-induced toxicity during screenings. We show the validity of this assay for a screening platform by testing two dietary phenolic acids, namely gallic acid (GA; 3,4,5-trihydroxybenzoic acid) and ferulic acid (FA; 4-hydroxy-3-methoxycinnamic acid), with well described anti-inflammatory actions in animal models of IBD. Analysis of common IBD therapeutics (Prednisolone and Mesalamine) proved the fidelity of our IBD-like intestinal inflammation model. In conclusion, the HCD-GI assay can facilitate and accelerate drug discovery efforts on IBD, by identification of novel lead molecules with immune modulatory action on intestinal neutrophilic inflammation. This will serve as a jumping-off point for more profound analyses of drug mechanisms and pathways involved in early IBD immune responses.

## Introduction

Inflammatory bowel diseases (IBD), like Crohn’s disease (CD), and ulcerative colitis (UC), are chronic inflammatory disorders of the gastrointestinal tract with significant morbidity and mortality worldwide (Burisch et al., 2013). IBD lacks a cure and affects *circa* 6.8 M people globally, bringing substantial costs to the healthcare system and society (Burisch et al., 2013; GBD 2017 Inflammatory Bowel Disease Collaborators et al., 2019). Although IBD incidence is increasing, disease onset is still unclear. The current view is that IBD arises from the intersection of multiple factors including genetic susceptibility, the intestinal microbiota, aberrant immune responses and environmental insults such as diet (GBD 2017 Inflammatory Bowel Disease Collaborators et al., 2019). This complexity makes the development of effective therapeutic strategies for IBD highly challenging. Coinciding with the occurrence of IBD, the rising consumption of diets high in fat, cholesterol, protein and sugar have been observed in the Western World (Molodecky et al., 2012). A systematic review of 19 studies reveals an association between Western-style diets and a higher risk of developing IBD (Hou et al., 2011). In mice and zebrafish, acute exposure to dietary cholesterol can induce acute innate inflammatory responses, with interleukin-1β (IL-1β)-dependent accumulation of myeloid cells in the intestine (Progatzky et al., 2014). This direct pro-inflammatory effect of ingested cholesterol occurs through inflammasome activation, involving Caspase-1 activity in intestinal epithelial cells for the localized production of IL-1β (Progatzky et al., 2014).

The present mainstay of IBD medical management involves anti-inflammatory drugs (prednisolone and mesalamine), immunomodulators (azathioprine and mercaptopurine), antibiotics and biological agents (Damião et al., 2019). However, none of these medications are curative, or free from having a high number of non-responders and significant side effects (Hossen et al., 2019). Consequently, there is an inevitable need to develop alternative therapeutic approaches to overcome these adverse events. As the innate immune system is known to initiate inflammation (Holleran et al., 2017), a promising therapeutic strategy is to target the innate immune axis at the initial stages of IBD. In fact, classic IBD therapies like mesalamine affect myeloid cell functions in multiple ways (Na et al., 2019). A common bottleneck to drug discovery and translational medicine is finding sufficiently accurate models that reproduce central aspects of disease development and progression. Traditional drug screening models often rely on simple two-dimensional (2D) culture systems that are very far from the complexity of the organs or the whole organism (Jardine et al., 2019). Three-dimensional (3D) tissue surrogates, like human intestinal organoids, have recently emerged as promising strategies to develop multiplexed screening platforms and to advance personalized medicine (Beaurivage et al., 2019). Although these systems may recapitulate the cellular diversity of the human intestinal epithelium, they lack immune cells which are key contributors to normal disease development (Sato et al., 2009; Beaurivage et al., 2019). Co-cultures of intestinal epithelial and immune cells have been explored in trans-well systems using transformed cell lines (Kämpfer et al., 2017); yet those are not sufficient to bridge the translational gap to the clinic. Recently, a 3D tissue system of the large intestine using human biopsy-derived colonic organoids and human primary monocyte-derived macrophages cultured in a 3D sponge scaffold has been developed to allow epithelial-immune interactions reflective of IBD (Roh et al., 2019). Although this system is promising as a lead validation platform, it turns to be quite complex for high- or even medium-throughput screening strategies and lacks the *in vivo* significance.

*In vivo* models of IBD have proven invaluable for the understanding of human intestinal diseases. Although, IBD mouse models are highly instructive platforms for investigating disease physiology, they are prohibitive for performing large-scale *in vivo* compound screens. Instead, zebrafish models of IBD provide a low-cost vertebrate model system for the initial stages of anti-inflammatory discovery programs (Brugman et al., 2009; Oehlers et al., 2011, 2012, 2017). Zebrafish larvae are especially amenable due to their small size, simple manipulation and observation, together with the fact that molecules can be added directly into their liquid media and are rapidly absorbed (Marjoram and Bagnat, 2015). Owing larval optical translucency and the availability of transgenic lines with fluorescently labeled myeloid cells (Lieschke et al., 2001; Buchan et al., 2019), zebrafish offers the unique advantage of monitoring an acute inflammatory response with non-invasive intravital or post-mortem imaging (Oehlers et al., 2012, 2017). This stands to be possible as major mammalian immune signaling mechanisms are considered to be conserved in zebrafish (Stein et al., 2007).

Several chemical-based models of enterocolitis have been adapted from the murine system into larval zebrafish. Zebrafish are especially amenable to chemical induction of gut inflammation, as larvae are simply bathed in the chemical incitant over time, allowing it to be swallowed and to create intestinal damage. Two high-content chemical screens on dextran sodium sulfate- (DSS) and trinitrobenzene sulfonic acid (TNBS)-induced zebrafish enterocolitis models have been performed and identified novel anti-inflammatory drugs suppressing neutrophilic inflammation (Oehlers et al., 2017). However, in DSS and TNBS models, severe toxicity was observed for a total of 14% of compounds (11% for DSS; 3% for TNBS) (Oehlers et al., 2017) and both chemicals induced extra-intestinal effects (e.g., leukocytosis) (Oehlers et al., 2011).

To circumvent these drawbacks, we report here a diet-targeted intestinal inflammation assay in zebrafish, that is highly physiological, easy to work and handle, reproducible and inexpensive. An improvement in the feeding strategy with high cholesterol diet (HCD) selectively induces a robust and consistent infiltration of myeloid cells in larvae intestines that is highly suitable for drug discovery efforts. The use of semi-automated image acquisition and processing combined with quantitative image analysis allows quantifying intestinal neutrophilic infiltration in transgenic zebrafish bearing fluorescently labeled neutrophils. Besides describing the assay as well as its optimization, we validate its applicability in a compound screening platform for the discovery of new immune modulatory molecules with relevance for IBD. Likewise, this assay is suitable for more in-depth analyses of drugs’ mechanisms of action; and may readily provide information on off-target effects at early phases of drug development.

## Materials and Equipment

### Transgenic Zebrafish

1. Tg(Lyz:NTR-mCherry)sh260 (Buchan et al., 2019), kindly provided by Stephen Renshaw lab.

### Materials

1. Polystyrene petri dishes (Thermo Scientific P5606-400EA).
2. Glass petri dishes (Fisher Scientific 11760834).
3. Eppendorf microloader pipette tips (Eppendorf 5242 956.003).
4. Disposable graduated transfer pipettes (VWR, 414004-036).
5. Glass pasteur pipettes 150 mm (FriLabo, 5426015N).
6. Beakers 5 mL (VWR 213-0010).
7. Disposable pestle (VWR KT749521-1500).
8. 250 mL sterile containers with lids (Corning 525-3408).
9. 1.5 mL microtubes (Abdos P10202).
10. 2 mL microtubes (Abdos P10203).
11. 12-well plates for tissue culture (VWR 10062-894).
12. 15 mL falcons (VWR 525-0604).
13. 50 mL falcons (Falcon 352070).
14. 10 mL Volumetric Pipettes (Costar Stripette 4488).
15. Pipette tips (Abdos P10102; Ahn myTip P-196816; Sarstedt 70.1131).
16. Aluminum foil (Trato Real).
17. Glass bottom culture dishes for microscopy (VWR 734-2906).

### Reagents/Solutions

1. Embryo Medium (E3) supplemented or not with methylene blue (see *recipes*).
2. Methylene blue (Sigma-Aldrich, M9140; 0.01% w/v prepared in dH_2_O).
3. NaCl (Fisher Scientific S/3120/65).
4. KCl (Merck Supelco 104936).
5. CaCl_2_.2H_2_O (Sigma-Aldrich, 223506).
6. MgSO_4_.7H_2_O (Sigma-Aldrich, M1880).
7. MS-222 (25X, 4000 ppm or mg/L; see *recipes*).
8. Dimethyl sulfoxide (DMSO; Sigma D5879).
9. PBS 1X (see *recipes*).
10. Na_2_HPO_4_ (Biochem Chemopharma 319360500).
11. KH_2_PO_4_ (Fluka 60229-1KG-F).
12. Distilled water (dH_2_O).
13. MilliQ H_2_O.
14. Cholesterol (Sigma-Aldrich C8667).
15. Diethyl ether (Sigma-Aldrich 296082).
16. Standard zebrafish larval food (Sparos Lda, Zebrafeed <100 μm; Analytical constituents: crude protein 63%, crude fat 14%, crude ash 12%, crude fibre 1.8%).
17. Caspase 1 Inhibitor I (Sigma-Aldrich 400010).
18. Gallic acid (Sigma-Aldrich G7384).
19. Ferulic acid (Sigma-Aldrich 128708).
20. Prednisolone (Sigma-Aldrich P6004).
21. 5-Aminosalicylic acid (5-ASA or mesalamine; Sigma-Aldrich A3537).
22. 4% w/v paraformaldehyde (PFA; Sigma, P6148) dissolved in PBS 1X (see *recipes*).
23. 1% w/v low gelling temperature agarose (Sigma-Aldrich A9414) dissolved in PBS 1X (see *recipes*).

### Equipment

1. Digital Incubator (VWR 390-0384).
2. Stereoscopes (Leica M125).
3. Fluorescence stereoscope (Zeiss Lumar V12).
4. Motorized and automated inverted microscope (Zeiss Axio Observer).
5. Micropipettes (Gilson).
6. Pipettor (Orange Scientific).
7. Vortex (Scientific Industries G560E).
8. Microcentrifuge (VWR, Galaxy MiniStar 0803-0298).
9. Dry block incubator [Eppendorf Thermomixer Comfort 5355 000.011 (European)].
10. Autoclave.
11. Chemical Hood.
12. Freezer (−20°C).
13. Fridge (4°C).

### Software

1. Zeiss Zen 3.0 (blue edition).
2. Huygens Software by Scientific Volume Imaging.
3. Image J 1.52c.
4. GraphPad Prism v.7.

### Recipes

1. Embryo Medium (E3)
  a. E3 stock (60X): Prepare 1 L by adding 17.20 g NaCl, 0.76 g KCl, 2.90 g CaCl_2_.2H_2_O and 4.90 g MgSO_4_.7H_2_O to 800 mL distilled H_2_O (dH_2_O). Mix well. Adjust the pH to 7.0. Add dH_2_O until a 1 L total volume. Autoclave and store at 4°C.
  b. E3 (1X): Dilute 16.7 mL of E3 60X stock in dH_2_O to make up 1 L. To supplement with the fungicide methylene blue, add 3.0 mL of 0.01% w/v methylene blue (0.01 g in 100 mL dH_2_O). Store at room temperature.
2. PBS
  a. PBS stock (10X): Prepare 2 L by adding 160.0 g NaCl, 4.0 g KCl, 28.8 g Na_2_HPO_4_ and 4.8 g KH_2_PO_4_ to 1.8 L MilliQ H_2_O. Mix well. Adjust the pH to 7.4. Add MilliQ H_2_O until a 2 L total volume.
  b. PBS (1X): Dilute 100 mL PBS 10X stock in 900 mL MilliQ H_2_O. Mix well and autoclave. Store at room temperature.
3. MS-222 (or Tricaine) solution
  a. Tricaine stock (25X): Prepare 500 mL at 15 mM (4000 ppm or mg/L) by adding 2 g MS-222 (Sigma-Aldrich E10521) and 10 ml Tris pH 9 to 400 mL dH_2_O. Adjust the pH to 7.0. Bring to 500 mL with dH_2_O. Store at 4°C.
  b. Tricaine (1X): To prepare the anesthetizing solution for the larvae, dilute the 25X stock in E3 or E3 supplemented with methylene blue to make up a 0.6 mM MS-222 (160 ppm or mg/L) final solution.
4. 4% paraformaldehyde (PFA) solution
  a. Add 4 g of PFA (Sigma, P6148) to 100 mL PBS 1X in the hood.
  b. Mix with heat and shake in magnetic plate until it is clear. Adjust the pH to 7.4. Aliquot in 2 mL tubes and store at −20°C.
5. 1% w/v low gelling temperature agarose
  a. Add 1 g low gelling temperature agarose (Sigma, P6148) to 100 mL PBS 1X. Heat until is clear. Aliquot in 2 mL tubes and store at 4°C.
  b. To be ready to use, heat the aliquot at 85–90°C and then keep it warm at 42°C.

## Methods

### High-Cholesterol Diet Gut Inflammation Assay – Establishment and Optimization

#### Animal Handling

For the high-cholesterol diet gut inflammation (HCD-GI) assay use 6 dpf larvae arising from group matings between heterozygous Tg(Lyz:NTR-mCherry)sh260 (Buchan et al., 2019). Collect embryos by natural spawning and raise them in polystyrene petri dishes at 28°C in E3 medium supplemented with 0.03% methylene blue as an antifungal agent. It is essential to maintain a density of embryos not exceeding 50 per petri dish. Great care should be taken to remove unfertilized eggs and chorions post hatching with a plastic Pasteur pipette under the stereoscope.

#### Larva Sorting

Check all larvae under a fluorescence stereoscope for homogeneous fluorescent reporter expression, spontaneous inflammation and appropriate age-related development. Larvae are sorted in fresh E3 medium supplemented with 0.03% methylene blue and MS-222 (1X; 160 mg/L). Larva sorting is preferred at about 3 to 4 dpf. If necessary, orient larvae in a lateral position using a flexible Eppendorf Microloader Pipette Tip for better visualization of fluorescent reporter expression. Place larvae in transparent 250 mL containers filled with 50 mL E3 medium supplemented with methylene blue until 6 dpf. Close the containers with holed lids and incubate them at 28°C.

#### Preparation of Cholesterol-Enriched Diet (HCD) and Control Diets (SP; SPE)

First weigh out 0.05 g cholesterol (Mw = 386.65 g/mol) and 0.45 g standard zebrafish larval food (SP; Sparos Lda; Zebrafeed <100 μm) and place in a 5 mL glass beaker to create a 10% w/w cholesterol-enriched diet, referred here as HCD. Components are homogenized and mixed by adding 1.5 mL diethyl ether that allows cholesterol solubilization. In another glass beaker, 1.5 mL diethyl ether is added to 0.5 g of SP to serve as a control diet (SPE; SP with diethyl ether). Diets are left overnight in the hood for the diethyl ether to evaporate completely and grounded up into fine particles using a pestle the following day.

#### 12-Well Plate Preparation

Pre-add 3 mL of freshly made E3 medium (without methylene blue) to each well with a 10 mL volumetric pipette. A glass Pasteur pipette is required to handle the embryos carefully without inflicting any wounding. Transfer single larva to each well in a minimum E3 volume for about twelve to fifteen larvae per well. Incubate the screening plate at 28°C. For media changes, add 3 mL of E3 medium to each well in a new 12-well plate and transfer larvae.

#### Feeding Strategy

Zebrafish larvae at 6 dpf are fed for 24 h with SPE control diet or cholesterol-enriched diet, HCD. Feeding occurs at three timepoints during the 24 h, with an interval of at least 6 h (see *protocol schematics in* Figure 2A; see *schedule below*). To avoid food deposition, media is replaced before the second feeding time. Food is added to each well with a micropipette tip to create a thin film at the medium surface. After the 24 h feeding period, larvae are transferred to fresh E3 medium (3 mL) where they are kept for 15 h without diet for intestine emptying until fixation. To avoid the presence of any food in the wells during the 15 h period, larvae are transferred twice to E3-filled petri dishes for wash outs and then placed in the wells.

**FIGURE 1:**
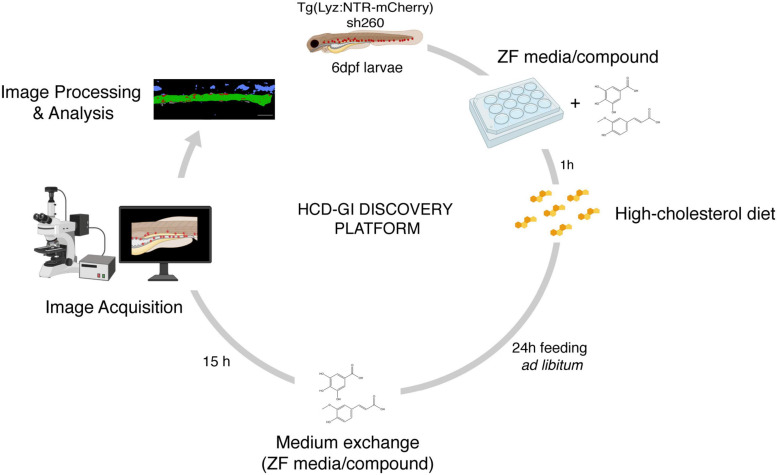
The high-cholesterol diet gut inflammation (HCD-GI) discovery platform. Schematic diagram illustrating the screening strategy for compounds modulating inflammation. Individual Tg(Lyz:NTR-mCherry)sh260 larvae, with fluorescent reporter expression in neutrophils, are distributed in 12-well plates with E3 zebrafish (ZF) medium supplemented with the compounds at 25 or 100 μM. After 1 h pre-treatment, larvae are fed with high-cholesterol diet (HCD) for 24 h. Larvae are then transferred to fresh medium supplemented with compounds were are kept for 15 h to allow inflammation and proper intestine emptying to occur. Larvae are finally fixed and imaged. Screening data are processed, and the neutrophilic inflammation index analyzed. Figure performed with images from Biorender.

**FIGURE 2:**
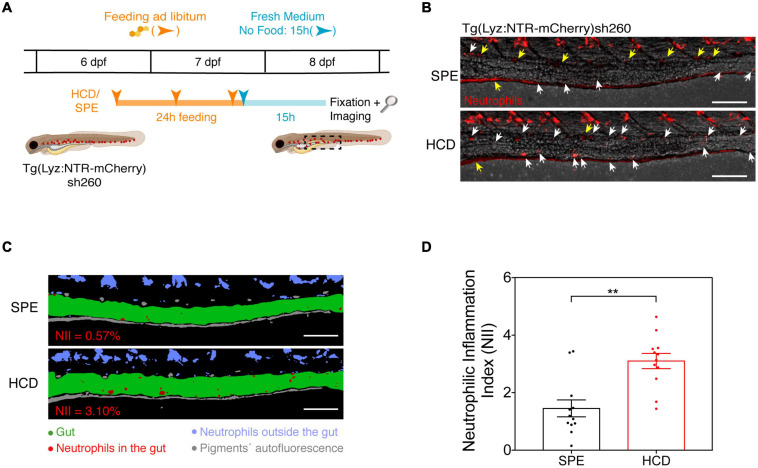
The HCD-gut inflammation (HCD-GI) assay. **(A)** Schematic representation of the protocol optimized for the HCD-GI assay in 6 dpf Tg(Lyz:NTR-mCherry)sh260 (Progatzky et al., 2014). **(B)** Representative images of distal intestine at 15 h following SPE or HCD feeding for a 24 h period. White arrows correspond to mCherry^+^ neutrophils in the intestines. Yellow arrows correspond to autofluorescence caused by pigments. **(C)** Image processing used for the quantification of neutrophilic inflammation index (NII) in larvae from **(B)**. NII values for the representative SPE or HCD larvae are shown in red. **(D)** NII quantification. One representative experiment of *n* = 12 larvae is shown. Each dot represents one larva. Two-tailed Mann Whitney test. **P < 0.01. Error bars represent SEM. Scale bars = 100 μm. Scheme **(A)** performed with images from Biorender.

Recommendations:

• The food amount that is provided to larvae is the minimum amount of powder that covers the medium surface (<1 mg). Procedure: 1- Dip a micropipette tip into the larval food so that the powder remains attached to the tip surface. 2 – Beat the tip against the well wall to free the particles into the medium surface.
• Schedule: Feeding 1: 5 pm (6 dpf) → Medium Exchange + Feeding 2: 10 am (7 dpf) → Feeding 3: 4 pm (7 dpf) → Medium Exchange: 5 pm (no diet; 7 dpf) → Fixation: 8 am (8 dpf).

**FIGURE 3:**
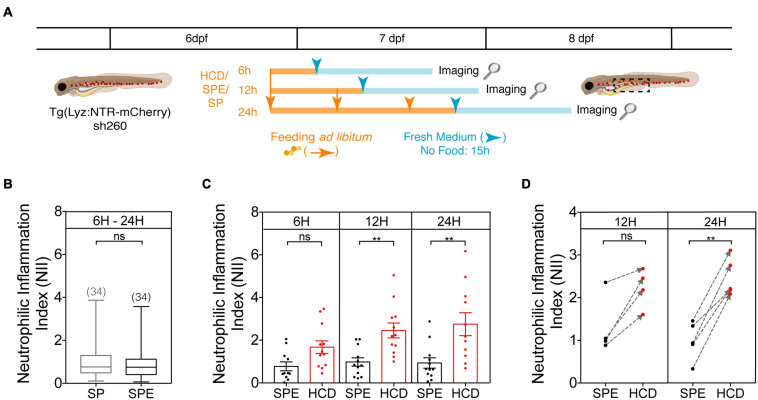
Protocol optimization for the HCD-GI assay. **(A)** Schematic representation of the optimization protocol used to establish the HCD-GI assay in 6 dpf Tg(Lyz:NTR-mCherry)sh260. **(B)** Box plots of mean neutrophilic inflammation index (NII) quantification at 15 h following feeding with standard zebrafish larval food (SP) or SP supplemented with the cholesterol solubilizer diethyl ether (SPE). Data were aggregated from one representative experiment of *n* = 10–12 larvae fed for 6, 12, or 24 h. Total number of averaged larvae is disclosed in parenthesis. Central lines represent median values, whereas box edges represent the 25 and 75th percentiles. **(C)** Neutrophilic inflammation index (NII) quantification at 15 h following HCD or control diet SPE feeding for a 6- (Progatzky et al., 2014), 12-, or 24 h period. One representative experiment of *n* = 10–12 larvae is shown. Each dot represents one larva. Error bars represent SEM. **(D)** Arrow plots of mean NII values. Mean NII of pools of *n* = 10–12 larvae were calculated for 4–5 independent experiments and represented by dots. SPE – Black dots; HCD – Red dots. The gray dashed arrows indicate the difference between mean NII in SPE- and HCD-fed animals in individual experiments. Two-tailed Mann Whitney test. **P < 0.01; n, non-significant. Scheme **(A)** performed with images from Biorender.

Note:

1. For the *Optimization of the HCD-GI Assay*, feed 6 dpf zebrafish larvae for 6, 12, or 24 h with HCD or control diets, SP or SPE (see *protocol schematics in* Figure 3A). Feeding occurs once for the 6 h, twice for the 12 h and three times for the 24 h, always with an interval of 6 h at least for multiple feedings. After the feeding period, larvae are kept for 15 h without diet until fixation.

#### Fixation

Larvae are anesthetized in E3 supplemented with tricaine (160 mg/L; E3/T) directly in the wells and transferred in a minimum volume to 2 mL microtubes containing 1 mL 4% PFA solution. Incubate larvae overnight at 4°C covered with aluminum foil to prevent them from losing fluorescence. Rinse them in PBS at least three times and store them in PBS at 4°C with aluminum foil until imaging.

#### Larvae Mounting and Imaging

Larvae are mounted lateral side down in 1% low gelling temperature agarose dissolved in PBS, over glass bottom culture dishes and overlaid with PBS for imaging. Use a flexible Eppendorf Microloader Pipette Tip to orient larvae in a lateral position for better visualization of fluorescent reporter expression. Z-stack acquisition is performed with Zeiss Zen 3.0 (blue edition) software for multipositions in an automated and motorized inverted microscope (Zeiss Axio Observer) using a 10× objective (NA 0.3) and a mercury lamp. Position the larvae to have the more distal part of the intestine centered anterior posteriorly and dorsal ventrally in the image. Set the lumen of the gut as the z-center level. Image each larva in the channels brightfield and mCherry (ex: 585 nm, em: 610 nm band pass filters) in 49 focal planes (149 μm range; 3 μm *z* step) so that all the gut depth is visible.

#### Image Processing and Analysis

##### Image deconvolution

Raw mCherry Z-stacks are deconvolved with the Huygens Essential 20.04 (Scientific Volume Imaging, Netherlands,^1^), using the classic maximum likelihood estimation (CMLE) algorithm, with signal to noise ratio (SNR) of 40, estimated background 250, 50 iterations and a quality threshold of 0.01. The software calculates a theoretical point spread function (PSF) to deconvolve the images. A wide variety of parameters were first tested and the ones used provided the best signal improvement without artifacts.

##### Quantification of the neutrophilic inflammation index

The quantification of the neutrophilic inflammation index (NII) was performed using Fiji (ImageJ) after image deconvolution (see *image processing and analysis pipeline in* Supplementary Figure 1; see *example in* Figures 2B,C). Maximum intensity projection (MIP) images from 49 focal planes are generated for each of the channels. Guts are manually drawn using the brightfield MIP image to create a gut region-of-interest (ROI). Care is taken to exclude pigments from the gut ROI. The mCherry MIP image is manually thresholded to create a mask for the red fluorescent leukocytes, reflected as red pixels in the image. Finally, the area fraction of the red pixels in the gut ROI is obtained with the *Measurement > Area Fraction* tool to calculate the percent area occupied by neutrophils in the defined area of the intestine, i.e., the NII.

##### Statistical analysis

Statistical analysis was performed in GraphPad Prism v7 and statistical significance was considered for *p* < 0.05. Comparison between samples was performed by a nonparametric Mann–Whitney test.

### Potential Applications

The new HCD-GI assay is highly physiological and suitable for translational, cellular or molecular applications, specifically:

(i) To develop a *drug-discovery platform with relevancefor IBD*. This assay can be exploited to test molecules for their effect on the initiation step of intestinal neutrophilic inflammation in the context of drug-repurposing or new compound screens;
(ii) To identify *dosage, tolerance, toxicity or off-target effects* of leads at early stages of drug development;
(iii) For *in-depth analyses of drug signaling mechanisms* involved in the orchestration of innate immune responses in IBD. The signaling pathways underlying HCD-induced intestinal innate inflammation are well characterized in zebrafish (Progatzky et al., 2014). These are a solid base for successive investigation of leads’ activity at a molecular level;
(iv) To identify *mutations affecting leukocyte migration* during intestinal inflammation.

### HCD-GI Discovery Platform

**FIGURE 4:**
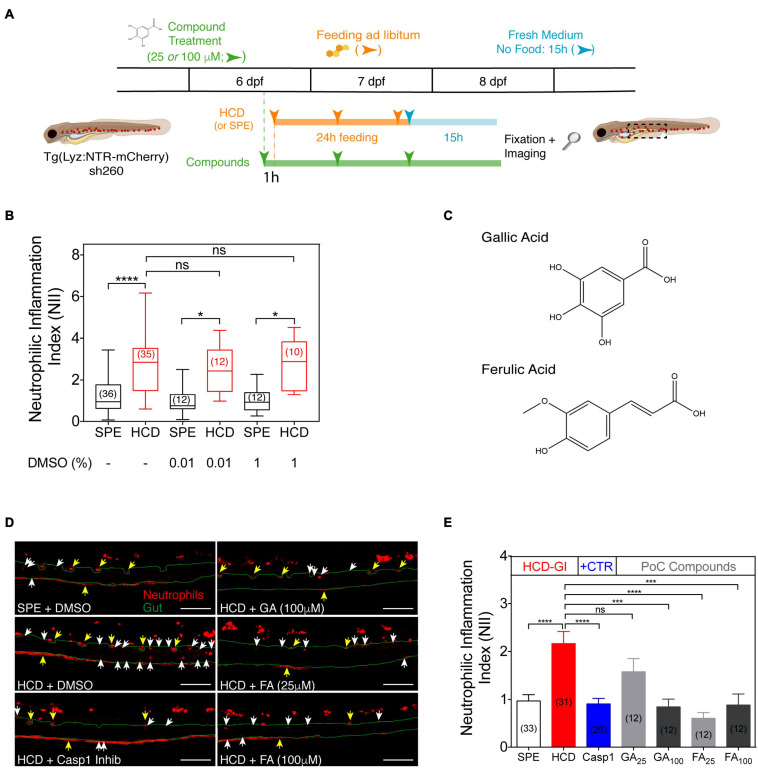
The HCD-GI discovery platform reveals an anti-inflammatory action of gallic acid (GA) and ferulic acid (FA) on zebrafish intestinal neutrophilic inflammation. **(A)** Schematic representation of the HCD-GI discovery platform in 6 dpf Tg(Lyz:NTR-mCherry)sh260. **(B)** Box plots of neutrophilic inflammation index (NII) analysis at 15 h following SPE or HCD feeding for a 24 h period in larvae untreated or treated with DMSO at different concentrations. Central lines represent median values, whereas box edges represent the 25 and 75th percentiles. Data were aggregated from 3 (DMSO: -) or 1 (DMSO: 0.01 or 1%) screening replicates and pools of n biologically independent animal experiments disclosed in parenthesis. **(C)** Chemical structures of GA and FA. **(D)** Representative images of distal intestine in GA, FA, or 1% DMSO-treated larvae. Caspase 1 Inhibitor I (Casp1) at 100 μM is used as positive anti-inflammatory control. White arrows correspond to mCherry^+^ neutrophils in the intestines. Yellow arrows correspond to autofluorescence caused by pigments. The intestine is outlined in green. Scale bars, 100 μm. **(E)** NII analysis in GA, FA, or DMSO-treated larvae. Data are presented as mean values ± SEM. Data were aggregated from 3 (SPE and HCD), 2 (Casp1), or 1 (GA and FA) screening replicates and pools of n biologically independent animal experiments disclosed in parenthesis. Kruskal–Wallis test (one-way ANOVA on ranks) was used for multiple comparisons. ****P < 0.0001; ***P < 0.001; *P < 0.05; ns, non-significant. Scheme **(A)** performed with images from Biorender.

We have developed a *drug-discovery platform with relevance for IBD (i)* – the HCD-GI discovery platform (*see schematics in* Figure 1 and Figure 4A) -, which can be used to test any soluble drug, signaling inhibitor, natural compound or small molecule. For a first-pass screening strategy, we suggest exposing the larvae to the compounds at two different concentrations (25 and 100 μM) throughout the whole assay. This is a good treatment option that increases the chance of identifying as many compounds as possible targeting an intestinal inflammation process at its initiation step. Based on the NII, the effects of the compounds can be evaluated at the end of the assay and categorized as anti- or pro-inflammatory. Importantly, Caspase 1 Inhibitor is used as an effective anti-inflammatory drug control of the screening platform whose molecular action is well-described (Progatzky et al., 2014). This inhibitor blocks IL-1β production in intestinal epithelial cells after HCD-exposure, a cytokine important for intestinal myeloid cells’ infiltration in this model (Progatzky et al., 2014).

Preclinical proof-of-concept of the platform is exemplified by testing the effects of gallic acid (GA) and ferulic acid (FA) on HCD-driven intestinal inflammation phenotype. GA and FA are active compounds found in many fruits and plants and exhibit anti-inflammatory protective effects in mouse models of IBD (Sadar et al., 2007; Pandurangan et al., 2015; Kandhare et al., 2016; Hossen et al., 2019; Zhu et al., 2019; Yang et al., 2020). After a first pass screening strategy, GA and FA are validated in dose-response experiments to find their IC50 and the lowest concentrations at which they are active. Notably, the clinical fidelity of our IBD-like platform is ultimately demonstrated by monitoring the effects of common IBD therapeutics, namely mesalamine and prednisolone (Oehlers et al., 2011, 2017), in NII values.

*Animal Handling, Larva Sorting, Preparation of Diets, Fixation, Imaging and Image Processing and Analysis* are performed as described in **HCD-GI Assay – Establishment and Optimization**.

#### Preparation of Compounds

First weigh out all the compounds under test (here exemplified for GA and FA) and the screening control (Caspase 1 Inhibitor I). Prepare 10 mM stock solutions in DMSO. Make aliquots to eliminate the need to expose compounds to repeated freeze/thaw cycling. Make DMSO aliquots for the negative and diet controls. Protect them from light and store at −20°C.

#### 12-Well Plate Preparation With the Compounds

Pre-add 2.970 or 2.992 mL of freshly made E3 medium to each well, in accordance with the volume of compound that will be added. Mix compounds in the stock aliquot three times by pipetting up and down or by vortexing. Add 30 μl (for 100 μM solution) or 7.5 μl (for 25 μM solution) compound stock to each well and mix medium in wells four times to ensure homogenous distribution of the compound within the well. Transfer single larva with a glass pipette to each well in a minimum E3 volume for about twelve to fifteen larvae per well. Incubate the screening plate at 28°C covered with aluminum foil to protect compounds from light. For media changes, use the same procedure.

Recommendation:

• Keep minimal the amount of medium that is added to the wells during larvae transfer to avoid diluting the screening solutions. Use glass Pasteur pipettes with a very small hole and let larvae accumulate in groups at their tips and swim directly to the medium.

#### Screening Controls

Treatment with 30 μl DMSO (or 1% DMSO; vehicle) in SPE-fed larvae is used as diet control. 1% DMSO in HCD-fed animals works as negative control. Caspase 1 Inhibitor I (30 μl; 100 μM solution) in HCD-fed animals is used as positive anti-inflammatory control (Progatzky et al., 2014).

#### Feeding and Treatment Strategies

Zebrafish larvae at 6 dpf are fed for 24 h with HCD in the presence of 25 or 100 μM compounds. Feeding occurs at three timepoints during the 24 h, with an interval of at least 6 h (see *protocol schematics in* Figure 1 and Figure 4A; see *schedule below*). The first feeding event occurs 1 h after larvae pretreatment with the compounds. To avoid food deposition and compound degradation, E3 media with 25 or 100 μM compounds is replaced before the second feeding time. After the 24 h feeding period, larvae are transferred to fresh E3 medium supplemented with the compounds at 25 or 100 μM where they are kept for 15 h without diet until fixation. To avoid the presence of any food in the wells during the 15 h period, larvae are transferred twice to E3-filled petri dishes for wash outs and then placed in the wells.

Recommendations:

• The food amount that is provided to larvae is the minimum amount of powder that covers the medium surface (<1 mg) (see **HCD-GI Assay** – *Feeding strategy*).
• After adding the food, let the larvae at room temperature with little light for 1 h. This ensures feeding to occur and avoids degradation of compounds with light. Then, incubate the screening plate at 28°C covered with aluminum foil until the next feeding or medium exchange event.
• Schedule: Treatment 1: 4 pm (6 dpf) → Feeding 1: 5 pm (6 dpf) → Medium exchange/Treatment 2 + Feeding 2: 10 am (7 dpf) → Feeding 3: 4 pm (7 dpf) → Medium exchange/Treatment 3: 5 pm (no diet; 7 dpf) → Fixation: 8 am (8 dpf).

#### Dose-Response Experiments and Clinical Validation

For dose-response experiments or clinical validation adapt the volumes of E3 and compounds appositely in *12-well plate preparation with the compounds* and in *HCD-GI discovery platform*. All stock solutions are prepared in DMSO at 10 mM concentration, apart from mesalamine, for which a 131 mM stock solution is prepared.

#### Statistical Analysis

Statistical analysis was performed in GraphPad Prism v7 and statistical significance was considered for *p* < 0.05. Non-parametric Kruskal–Wallis test (one-way ANOVA on ranks) was used for multiple comparisons. The half maximal inhibitory concentration (IC50) was calculated based on a nonlinear regression using a four-parameter variable slope.

## (Example) Results

### The HCD-GI Assay

The HCD-GI assay is a simple, reproducible, economic and physiological model of innate intestinal inflammation with powerful applicability for a compound discovery platform in the whole organism system (Figure 1). Neutrophilic distribution is a generally used live readout of inflammation in larval zebrafish enterocolitis models (Oehlers et al., 2017). Under physiological conditions, neutrophils are primarily located in the caudal hematopoietic tissue (CHT) in the zebrafish larvae (Guyader et al., 2008). Exposure to chemical or environmental gut incitants causes a qualitative shift in neutrophil localization to the intestine (Oehlers et al., 2011; Progatzky et al., 2014). Examination of this phenotype for medium or high-throughput and high-content (HT/HC) efforts allows the identification of compounds exacerbating inflammation (pro-inflammatory) or inhibiting it (anti-inflammatory). Here we use dietary cholesterol as a highly physiological trigger of intestinal inflammation in zebrafish larvae (Progatzky et al., 2014). In contrast to chemically induced zebrafish enterocolitis models, dietary cholesterol targets inflammation selectively to the intestine when added acutely (Progatzky et al., 2014) and the chances of compound-induced toxicity are low.

Prior reports have revealed that zebrafish larvae fed for 6 h with dietary cholesterol develop acute innate inflammation with myeloid cell infiltration in the intestines (Progatzky et al., 2014). Significant differences in myeloid cell accumulation were only found using doses of 4% cholesterol with groups of about 30 larvae, and power calculations determined significant differences for 2% cholesterol when using *n* = 190 (Progatzky et al., 2014). Since these numbers are too high for a screening platform, we here develop an improved feeding strategy with more robust and adequate immune responses for this type of application.

In the HCD-GI assay, 6 dpf larvae are fed *ad libitum* three times during a 24 h period with 10% w/w cholesterol-enriched diet (HCD) or control larval diet (SPE; prepared as HCD but without cholesterol addition; *see recipes*) (Figure 2A). The assay is performed in Tg(Lyz:NTR-mCherry)sh260 larvae, where neutrophils are marked by the expression of monomeric Cherry (mCherry) (Buchan et al., 2019) (Figure 2A). We monitor the presence of mCherry^+^ neutrophils in larval intestines 15 h post feeding (Figure 2B). We choose this timepoint of observation for two main reasons: (1) To ensure perfect intestine emptying after feeding; (2) A peak of inflammation has been proposed to occur 15 to 18 h post HCD feeding and to resolve at about 24 h (Progatzky et al., 2014). Brightfield and mCherry images are collected and the quantification of NII is performed using Fiji (ImageJ) after image deconvolution. Briefly, the gut area is manually drawn. Then, a threshold is applied to the mCherry image to reduce the operator bias (Image – Adjust – Threshold). Finally, the area fraction of the mCherry pixels in the intestine is obtained with the *Measure* tool *(Set Measurements > Area Fraction)*, and this value corresponds to the NII (Figure 2C and Supplementary Figure 1). Taking into account the intrinsic variability in food intake by larvae during feeding we show the robustness of our protocol using a sample size *n* of about 12 larvae. We observe a strong inflammatory response in HCD-fed animals compared to SPE controls using groups of only 10 to 12 larvae (Figure 2D). These numbers are perfectly compatible with screening strategies.

### Protocol Optimization

For prior protocol optimization, a range of feeding times – 6, 12, and 24 h – were tested to establish the time that causes the strongest and most consistent inflammation with HCD (Figure 3A). 6 dpf larvae were fed *ad libitum* for 6 to 24 h with HCD or control larval diets. As control larval diets we tested standard zebrafish larval food (SP) or SP supplemented with diethyl ether (SPE), the organic solvent that helps solubilizing cholesterol in the cholesterol-enriched diet, HCD. At first, we looked whether the diethyl ether could non-specifically induce an inflammation phenotype. To this aim, we compared the inflammation index NII in SPE and SP groups aggregating the data from all feeding times. No significant differences were detected between these control groups (Figure 3B). This suggests that the phenotype observed in HCD-fed animals is highly specific to cholesterol enrichment and discards any effect of the organic solvent in the observed inflammation phenotype. Since the diethyl ether is left to evaporate overnight from the diet this result is expected. Thus, we use SPE as the proper control diet in the HCD-GI assay. Secondly, by comparing HCD-fed animals with SPE controls for the different feeding times, significant differences in the inflammation index occur after 12 or 24 h feeding using groups of 10 to 12 larvae (Figure 3C). Still, we favor the 24 h versus the 12 h feeding strategy for the HCD-GI assay because: (1) The strength of inflammation in HCD-fed animals relative to SPE controls is comparably higher (Figures 3B,C); (2) The increase of inflammation observed in HCD-treated animals respect to SPE controls is more consistent and reproducible (Figure 3D); (3) The success of individual experiments is a mandatory factor for a reliable discovery platform (Figure 3D).

### Applications: The HCD-GI Discovery Platform

To demonstrate the potential of our model for screening strategies, we developed the HCD-GI discovery platform (Figure 4A). In our platform, the HCD-GI assay is performed in the presence of the compounds throughout all the procedure. We opt for a prolonged compound treatment to increase the chances of identifying molecules with putative actions at the onset of intestinal inflammation. We also choose to employ the compounds at two different concentrations to avoid an erratic exclusion of compounds with no activity at the lowest one. With this strategy we can also find an initial range of action for each compound, to use in successive dose-response experiments. Therefore, we test a concentration commonly used in zebrafish drug screens - 25 μM (Rennekamp and Peterson, 2015) -, and also a higher concentration – 100 μM -, that turns to be useful when testing natural compounds with low toxicity and, possibly, lower activity (Figure 4A).

For the HCD-GI discovery platform, we use a sample size *n* of 12 to 15 larvae, and we prepare duplicates of control samples to provide robustness to the data. Since all compound stocks are dissolved in DMSO, we first determined if the concentration of the vehicle could non-specifically generate an inflammatory phenotype. In a dose-response analysis ranging from 0.01–1% DMSO, concentrations up to 1% have no effect in the neutrophilic inflammation index, NII (Figure 4B). Being 1% DMSO the highest concentration present in the liquid media of the animals under test (when exposed to compounds at 100 μM), we use it as a vehicle control in HCD- and SPE-fed larvae (*see samples below*). On the other hand, we use Caspase-1 inhibitor as the anti-inflammatory drug control of the platform, whose molecular action blocking HCD responses is well-known (Progatzky et al., 2014).

In summary, samples in the platform are:

(i) Control diet: SPE + 1% DMSO.
(ii) Negative control: HCD + 1% DMSO.
(iii) Anti-inflammatory drug control: HCD + Caspase 1 Inhibitor I (100 μM final concentration).
(iv) *n* compounds under test: HCD + *n* compounds (25- or 100 μM final concentration).

As a proof-of-concept for the HCD-GI discovery platform, we test two natural compounds, namely gallic acid (GA) and ferulic acid (FA), with known antioxidant and anti-inflammatory actions in mouse models of IBD (Figure 4C; Chaudhary et al., 2019; Kahkeshani et al., 2019). As expected, HCD-fed larvae show a strong inflammatory response with respect to the SPE counterpart. This inflammatory response is efficiently reduced to basal levels in the presence of Caspase-1 inhibitor, the anti-inflammatory drug control of the platform (Figures 4D,E). Interestingly, the dietary phenolic acid GA reveals an anti-inflammatory action at 100 μM comparing to HCD-fed animals (Figures 4D,E). Notably, FA shows a comparably more potent anti-inflammatory activity, strongly reducing NII at 25 μM (Figures 4D,E).

### Applications: Dose Response Analyses and IBD Therapeutics

**FIGURE 5:**
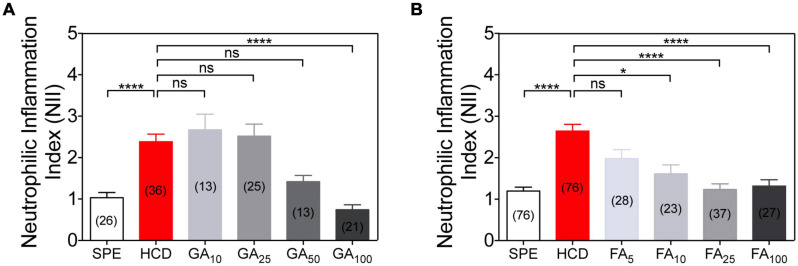
Dose-response anti-inflammatory action of gallic acid (GA) and ferulic acid (FA) using the HCD-GI assay. **(A)** Neutrophilic inflammation index (NII) in DMSO or GA-treated larvae. Data were aggregated from 3 (HCD), 2 (SPE, GA25, and GA100) or 1 (GA10 and GA50) experimental replicates and pools of *n* biologically independent animal experiments disclosed in parenthesis. **(B)** NII in DMSO or FA-treated larvae. Data were aggregated from 6 (SPE and HCD), 3 (FA25 and FA100) or 2 (FA5 and FA10) experimental replicates and pools of *n* biologically independent animal experiments disclosed in parenthesis. Data are presented as mean values ± SEM. Kruskal–Wallis test (one-way ANOVA on ranks) was used for multiple comparisons. ****P < 0.0001; *P < 0.05; ns, non-significant.

Successive dose-response analyses with GA and FA allow validating these results as well as identifying the lowest concentration at which these compounds are active and their IC50 (Figures 5A,B). Both compounds reveal an increased anti-inflammatory action with increasing molecular concentrations (Figures 5A,B). GA is only anti-inflammatory at 100 μM based on NII values (Figure 5A), having a IC50 of 44.13 μM. We also confirm that FA is a highly potent active molecule. It evokes an anti-inflammatory response in the HCD-GI model at concentrations as low as 10 μM (Figure 5B), having a half maximal inhibitory concentration (IC50) of 8.10 μM. Importantly, FA shows some toxicity at 100 μM, with certain larval suffering or death.

**FIGURE 6:**
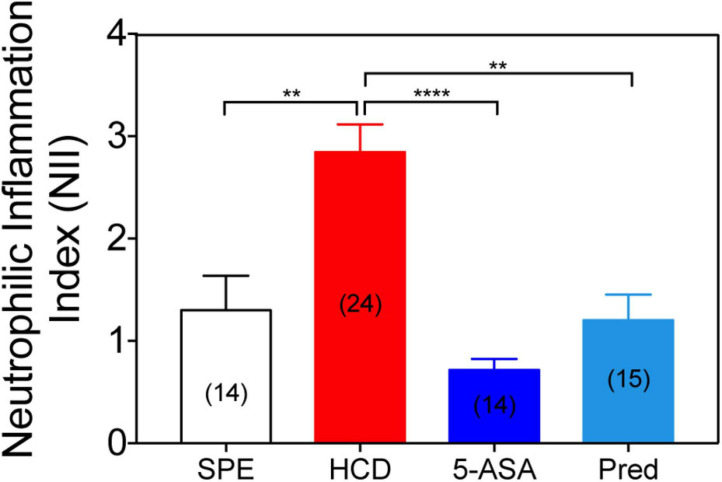
Treatment with conventional IBD therapeutics reduces the neutrophilic inflammation index. Neutrophilic inflammation index (NII) analysis in prednisolone (25 μM), mesalamine (5-ASA; 0.33 mM) or 1% DMSO-treated larvae. Data were aggregated from 2 (HCD) or 1 (SPE, Prednisolone, and 5-ASA) experimental replicates and pools of *n* biologically independent animal experiments disclosed in parenthesis. Data are presented as mean values ± SEM. Kruskal–Wallis test (one-way ANOVA on ranks) was used for multiple comparisons. ****P < 0.0001; **P < 0.01; ns, non-significant.

To illustrate the effectiveness of the HCD-GI platform in identifying immunomodulatory molecules with relevance for IBD, we evaluate the effect of common IBD therapeutics (prednisolone and mesalamine or 5-ASA) on the NII phenotype (Oehlers et al., 2011). The prescribed IBD drugs were employed in the platform at concentrations equal or lower to the ones reported in larval zebrafish enterocolitis models (mesalamine = 0.33 mM; prednisolone 25 μM) (Oehlers et al., 2011). Treatment with mesalamine reveals a strong anti-inflammatory action when compared to HCD-fed animals. NII levels in mesalamine-treated larvae are even below the diet control SPE (Figure 6). In basal conditions, commensal microbiota stimulates neutrophil migration and infiltration in zebrafish larval intestines (Kanther et al., 2014). It is possible that mesalamine exerts an anti-microbial effect impairing the homeostatic recruitment of immune cells into the intestine (Kaufman et al., 2009). On the other hand, administration of prednisolone was successful at reducing the recruitment of leukocytes to the intestine of HCD-fed larvae up to basal NII levels. These results validate the fidelity of the IBD-like neutrophilic inflammation phenotype in our model and its suitability for testing clinical grade molecules.

### Further Applications and Advantages

Apart from monitoring immune cells infiltration, several techniques can be applied to the HCD-GI assay to deeply investigate the underlying mechanisms of a drug discovery portfolio. A panel of anti- (e.g., IL-4 and IL-10) and pro-inflammatory [e.g., IL-1β and IL-6, Tumour Necrosis Factor-α (TNFα)] cytokines can be analyzed by quantitative polymerase chain reaction (qPCR) in intestinal cells or by enzyme-linked immunosorbent assay (ELISA) of the intestines’ supernatant. Moreover, the levels of reactive oxygen species (ROS), TNFα, IL-1β and Nuclear Factor kappa B (NF-κB)-signals, known to be upregulated in HCD-driven inflammation (Progatzky et al., 2014), could be singly monitored by using specific transgenic lines fed with HCD (Kanther et al., 2011; Enyedi et al., 2013; Marjoram et al., 2015; Ogryzko et al., 2018). On the other hand, leads can be validated in diet-based murine models of IBD (Progatzky et al., 2014) or in more humanized 3D tissue systems of the large intestine mimicking epithelial immune interactions reflective of IBD (Roh et al., 2019). This would allow validating the human relevance of leads.

### Pitfalls and Troubleshooting

The HCD-GI assay is highly sensitive and selective and is based on an unbiased quantification system (Figures 2B,C and Supplementary Figure 1). Being the most error-inducing step of the analysis performed manually, the operator can check the data blindly and analyze the results for errors. One of the confounding factors is the presence of red autofluorescence in the pigmented cells that are localized close to the intestine. If not excluded properly, those pigments introduce artifacts in the NII value, overestimating it. On one side, we can opt to employ phenylthiourea (PTU) during larval development, a potent tyrosinase inhibitor commonly used to suppress pigment formation in zebrafish. Yet, PTU can introduce another level of complexity to the system, as this chemical can potentially interfere with the compounds under test. We bypass this pitfall by manually drawing the intestinal area and excluding the pigments from the NII quantification (Figures 2B,C and Supplementary Figure 1). Still, it is important to avoid the introduction of bias from the operator in this step. Manual analysis should then be performed by two independent and highly trained operators.

Another limitation of the method is that although the quantification procedure is very sensitive it is manual. This can be easily circumvented to allow a fully automated image analysis suitable for high-throughput and high-content (HT/HC) screens. In that case, we suggest using in the platform double transgenic animals bearing fluorescently labeled gut epithelial cells and neutrophils (Oehlers et al., 2011). This would allow the automatic signal detection and quantification of neutrophil infiltration in larval intestines using the intestine-neutrophils colocalization pixels as an inflammation index (Wittmann et al., 2012, 2015).

A possible error inducing step in the HCD-GI platform is to manually place larvae in wells after the 25 or 100 μM solutions are already prepared. This procedure may dilute the testing solutions further. We minimize this error, using glass Pasteur pipettes with a very small hole to transfer larvae. With these pipettes, larvae often accumulate in groups at their tips and swim directly to the medium without the need to add extra volume to the wells. Like this, we can keep minimal the amount of medium that is added during larvae transfer. Also, this problem is bypassed by performing the screen at two different concentrations, which allows a preliminary titration that can be further validated in dose-response analyses. On the other hand, the same error would occur if larvae were placed in wells before adding the compounds; and in that case, the stock compound solution prepared in 100% DMSO would enter in direct contact with larvae in the wells, which can be very aggressive and should be avoided. For all these reasons we suggest to first dilute and mix the compounds in the medium before exposing the larvae to the testing concentrations. Notably, with robotic liquid handling of larvae this error can be circumvented (Wittmann et al., 2012, 2015).

Finally, as in previously reported screening approaches (Wittmann et al., 2012, 2015; Oehlers et al., 2017), we opted for a prolonged compound treatment to ensure the identification of all the compounds with possible immunomodulatory action in our setting. This option, however, does not allow to discriminate at which level compounds are acting or if they have a preventive or therapeutic potential. Still, after a first-pass screen, hits can be further tested to address how they are protective against HCD-induced intestinal inflammation. For instance, even if protection would occur at the level of cholesterol uptake this beneficial effect could be exploited as a preventive strategy in equivalent human exposure scenarios.

## Discussion

Dietary cholesterol-induced intestinal inflammation in larval zebrafish is a highly physiological model that recapitulates hallmark aspects of human IBD. In acute settings, these include the induction of pro-inflammatory pathways and degradative enzymes and the infiltration of innate immune cells in the intestines (Progatzky et al., 2014). After prolonged feeding schemes it leads to functional dysregulation as well as systemic pathologies (Progatzky et al., 2014). In Progatzky et al., significant differences in myeloid cell accumulation occurred with groups of more than 30 larvae fed with HCD. These numbers of larvae turn to be high and render screening strategies quite laborious. Here we report an optimized version of this model that is highly suitable for a drug-discovery platform in a whole organism context. By manipulating the dietary strategy to 24 h feeding, the HCD-GI assay reveals a robust degree of neutrophilic infiltration with groups of *circa* 12 larvae that is highly consistent between experiments (Figure 2 and Figures 3C,D). Neutrophilic infiltration is measured with a standardized protocol based on image deconvolution and analysis of an intestinal inflammation index, NII (Figures 2B,C and Supplementary Figure 1). Translating the HCD-GI assay into a discovery platform – the HCD-GI discovery platform (Figure 1) – allows the identification of immune-modulatory compounds of acute neutrophilic inflammation with potential relevance for IBD.

There is a clear unmet need to find new efficient treatments for IBD. This protocol was devised to apply zebrafish as a valuable *in vivo* model for compound discovery efforts, as the effects of molecules acting on intestinal inflammation can be straightforwardly studied in the context of an entirely functional innate immune system. A powerful aspect of our set-up is the use of transgenics highlighting the innate immune cell compartment, namely neutrophils, that allows monitoring the pathophysiology of an acute inflammatory response *in vivo*. Besides, a unique advantage of our method is the fact that an intestinal-targeted damage via dietary intake renders manual larvae manipulation minimal, thus permitting the screening in medium or high-throughput formats (Oehlers et al., 2011; Wittmann et al., 2012, 2015). Moreover, adverse and toxic effects of compounds and off-target outcomes can be evaluated in our platform, as compounds’ toxicity usually leads to larval deformities or death. In that case, it is possible to perform dose-response experiments over a range of well tolerated concentrations. This turns to be more relevant when the toxic compound belongs to a family of molecules that is known to exert promising immune modulatory actions.

Two high-content small molecule screens on DSS- and TNBS-induced enterocolitis have been reported in larval zebrafish (Oehlers et al., 2017). These chemicals induce colitis either by direct toxicity on epithelial cells or by disruption of intestinal barrier integrity. One of the drawbacks of using these agents in drug-screening assays are their extra-intestinal effects (Oehlers et al., 2011). As DSS or TNBS are added directly to the water medium, the route of exposure is not controlled and may lead to potential interactions with any epithelial surface and consequent skin damage (Oehlers et al., 2011). For instance, a relevant extra-intestinal consequence of TNBS treatment in larval zebrafish is the induction of leukocytosis (Oehlers et al., 2011). Although leukocytosis is a recognized hallmark of IBD, it is not clear how TNBS is inducing the redistribution of neutrophils from CHT region to the periphery. Another disadvantage is that these chemicals may react with the compounds under test (Oehlers et al., 2017). This may lead to toxicity or may provoke the tested compounds to lose their activity along the assay (Oehlers et al., 2017). As said, using diet as an incitant of acute intestinal inflammation avoids all the pitfalls underlying DSS or TNBS immersion, as is a more controlled, targeted, selective, and physiological trigger.

Similar to Oehlers et al. (2017) or other screens capable of identifying immunomodulatory drugs (Wittmann et al., 2012, 2015), we here present a screening strategy based on a prolonged compound treatment. This strategy ensures that all the compounds with immunomodulatory functions on acute intestinal inflammation are identified and avoids excluding putative candidates at early stages of a discovery plan. After a first-pass screen, hits should be validated and further investigated for their preventive or therapeutic action. By modifying duration and timing of compound application in the HCD-GI discovery platform, it is possible to discriminate whether molecules have a more preventive or treatment potential. For example, the dietary phenolic acids like FA or GA have a huge preventive and nutraceutical potential besides their possible pharmacological applications; and, nowadays, the ability to modulate diet to prevent daily an IBD condition is a highly attractive and an ever-demanding proposition for increasing quality of life.

Previous drug-repurposing screens in larval zebrafish enterocolitis models employed only qualitative observational identification of screening hits (Oehlers et al., 2017). We improved this approach by developing a semi-automated image acquisition and processing combined with quantitative image analysis for accurate quantification of NII. The intestinal area and red-labeled neutrophils are imaged 15 h post feeding, when intestines are perfectly emptied and the inflammation peak is most likely occurring (Progatzky et al., 2014). Red-fluorescent images of neutrophils are deconvolved calculating a theoretical Point-Spread-Function (PSF) and parameters are carefully chosen to provide the best signal improvement without artifacts. Manual drawing of the intestines permits calculating the red-fluorescent area of neutrophils inside intestines, the NII parameter, while correcting for pigments’ autofluorescence that must be excluded from the analysis (Figures 2B,C and Supplementary Figure 1). This parameter allows the analysis of inflammation through the application of an intensity threshold that reduces the operator bias and avoids the more laborious calculation of infiltrated cell numbers. Similar analysis has been performed for an automated high-content inflammation assay in zebrafish (Wittmann et al., 2012, 2015).

An option to make the HCD-GI discovery platform valuable for high-throughput and high-content (HT/HC) screens is to perform the protocol in double transgenic larvae highlighting both the intestinal enterocytes and the neutrophils (Oehlers et al., 2011). This would allow automatic signal detection and fully automated quantification of intestine-neutrophils colocalization pixels as an index of intestinal inflammation (Wittmann et al., 2012, 2015). Besides, this would automatically eliminate artifacts introduced by red autofluorescent pigments, as these would not colocalize with the intestinal labeling.

Intestinal neutrophilic infiltration is the readout system of the HCD-GI assay and platform. While the role of neutrophils in IBD has been investigated in animal models, their contribution to disease pathogenesis remains controversial and no molecules targeting neutrophils are validated for IBD treatment (Wéra et al., 2016). Controversy arises from the dual roles of neutrophils in inflammation. Neutrophils can be either beneficial for bacterial clearance and resolution of inflammation or detrimental when over-activated, leading to collateral tissue damage. It is therefore proposed that both functional deficiency and hyper-reactivity of neutrophils can induce intestinal inflammation in IBD (Wéra et al., 2016). For example, in UC the degree of neutrophils infiltration in the colon as well as their increased activity have been linked to the severity of the disease (Raab et al., 1993; Demir et al., 2015). Conversely, neutrophil dysfunction has been observed in CD patients (Segal and Loewi, 1976; Harbord et al., 2006). Above this duality, a powerful feature of our platform is the possibility to categorize compounds as anti- or pro-inflammatory when, respectively, reducing or increasing the NII inflammation index (Figure 4). Compounds belonging to each one of the categories can be further validated in independent experiments, as they might exert their effect in a dose-dependent manner (Figure 5). We could also expand the applicability of the HCD-GI platform to identify compounds affecting the resolution of neutrophilic inflammation. In that case, the timeframe at which resolution occurs should be monitored, which is between 18 and 24 h post feeding for a 6 h feeding strategy (Progatzky et al., 2014). Interestingly, the presented technique could also offer the possibility to be utilized for the identification of mutations in genes affecting neutrophil migratory behavior. Finally, from a molecular point of view, identifying new molecules acting on neutrophil infiltration capacity will improve our understanding of their mode of action and ultimately provide new therapeutic avenues that urge for IBD.

The zebrafish is an organism widely used in inflammation biology. The anti-inflammatory activity of food compounds has been already assayed in this model to evaluate their protective immunomodulatory functions (Arteaga et al., 2018). For the proof-of-concept of the platform, we here use two dietary phenolic acids, namely gallic acid (GA) and ferulic acid (FA), with well-described anti-inflammatory and antioxidant properties in murine models of IBD (Sadar et al., 2007; Pandurangan et al., 2015; Kandhare et al., 2016; Zhu et al., 2019; Yang et al., 2020). Indeed, we verified that both compounds reduced the inflammation index NII in animals after the induction of inflammation with HCD (Figures 4D,E). They exerted their effect in a dose-dependent manner, being FA more potent (Figures 5A,B). We also validated our IBD-like model using conventional anti-inflammatory IBD therapeutics, such as mesalamine and prednisolone (Figure 6A). The fidelity of our model was confirmed as both drug indications reduced the NII to homeostatic levels or below (Figure 6A). Altogether, these results suggest that the anti-inflammatory capacity of compounds can be evaluated with the HCD-GI discovery platform, and that the protocol creates a physiologically relevant environment for screening of clinical grade immune modulators for IBD.

In conclusion, the optimized HCD-GI assay serves as a simple, physiological, innovative, cost- and time-saving strategy to ease and accelerate drug discovery, or drug repurposing efforts, by providing potential new lead molecules relevant for IBD. This will serve as a jumping-off point for more profound analyses of drug mechanisms and pathways involved in the disease innate immune responses. Finally, using this assay at early phases of a drug discovery pipeline may provide readily available information on drug tolerance, toxicity, dosage as well as potential off-target effects that will reduce action risks and costs during drug development.

## Data Availability Statement

All datasets generated for this study are included in the article/Supplementary Material.

## Ethics Statement

The animal study was reviewed and approved by Animal User and Ethical Committees at Centro de Estudos de Doenças Crónicas (CEDOC) – NOVA Medical School and the Portuguese National Authority for Animal Health (DGAV).

## Author Contributions

N-VS conducted all the experiments, analyzed, and performed the data analysis, and statistical analysis with the help of CC, DC, and CG. AJ, CS, OV, and DC contributed to scientific discussion and revised the manuscript. CC conceived and supervised the study with the help of AJ and CS, designed experiments, interpreted the data, assembled the figures, and wrote the manuscript. All authors critically reviewed the manuscript and approved the submitted version.

## Conflict of Interest

The authors declare that the research was conducted in the absence of any commercial or financial relationships that could be construed as a potential conflict of interest.

## Footnotes

1 http://svi.nl

## Supplementary Material

Supplementary Figure 1 Image processing and analysis pipeline for quantification of the neutrophilic inflammation index (NII). Scheme performed with images from Biorender.
