# Supplementary figures and images for: A Dietary Cholesterol-Based Intestinal Inflammation Assay for Improving Drug-Discovery on Inflammatory Bowel Diseases
Source: Front Cell Dev Biol. 2021 Jun 3;9:674749. doi: 10.3389/fcell.2021.674749 (PMC8209420; doi:10.3389/fcell.2021.674749)

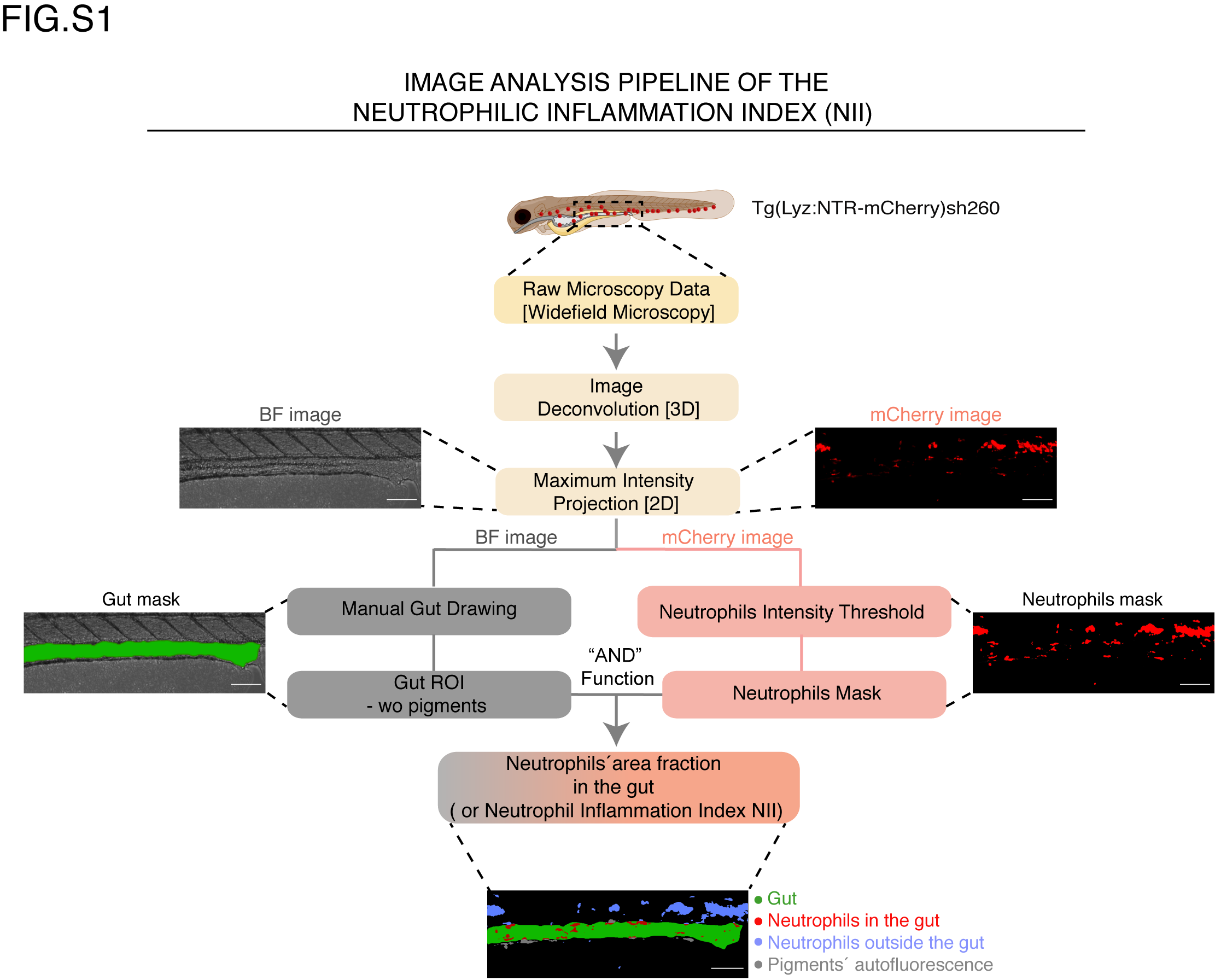

Supplement: Supplementary Figure 1 — Image processing and analysis pipeline for quantification of the neutrophilic inflammation index (NII). Scheme performed with images from Biorender. [file Image_1.TIF]
